# Reconsidering children’s illness uncertainty and management experiences with female Japanese cancer survivors

**DOI:** 10.3389/fpsyg.2022.1006267

**Published:** 2022-11-23

**Authors:** Yu Ishii, Toshihiko Endo

**Affiliations:** ^1^The Center for Early Childhood Development, Education, and Policy Research (CEDEP), Graduate School of Education, The University of Tokyo, Tokyo, Japan; ^2^Graduate School of Education, The University of Tokyo, Tokyo, Japan

**Keywords:** illness uncertainty, uncertainty management, pediatric illness, pediatric cancer, survivors

## Abstract

The aim of this study is to examine the illness uncertainties (IU) that children experience and the ways they manage them in order to construct a long-term, comprehensive developmental care for children with illness. Semi-structured, in-person interviews were conducted with six Japanese female adolescent cancer survivors, all recruited from the same hospital in Tokyo, Japan. Using directed content analysis, all transcriptions that fell under the definition “the person is unable to construct the meaning of an illness related event of her/his self or another, and is aware of the state” were coded with the codes defined from the data, which were then cross-referenced with the existing literature. The results indicated new aspects of children’s IU; uncertainties regarding hospitalization and the necessity to distinguish and examine IU with and without answers. In terms of IU management, we discuss the informative value “role models,” and depict how uncertainty acceptance may be a new form of solution.

## Introduction

In Japan, the long-term survival rate of childhood cancer and other illnesses that were once considered fatal has improved dramatically with the significant advance of medical treatment (e.g., Takahashi et al., 2018). However, even now, psychological care provided in hospitals for children are aimed primarily to reduce the distress children are experiencing at that moment, and despite the strong demand (Omata, 2018), no care has been developed with a long-term developmental perspective in mind. In this study, we focused on illness uncertainty (IU) as a potential key concept for constructing long-term, comprehensive developmental care for children with illness.

IU is defined by Mishel (1988) as “the inability to determine the meaning of illness-related events when a patient cannot structure or categorize an event because of insufficient cues.” The concept has attracted great attention from researchers who seek to improve the quality-of-life of patients with various illnesses. Previous research has dealt with patients of wide age range worldwide and it is now known that higher levels of IU directly correlate with higher levels of depression, anxiety, and poorer quality of life (e.g., Northouse et al., 1995; Sammarco, 2001; Clayton et al., 2006; Giurgescu et al., 2006; Szulczewski et al., 2017).

No doubt, the development of the Child Uncertainty in Illness Scale (CUIS; Mullins and Hartman, 1995; Pai et al., 2007) and the Uncertainty Scale for Kids (USK; Stewart et al., 2010) have facilitated research in the context of pediatric illness. Similar to that of adult research findings, Szulczewski et al.’s (2017) meta-analysis revealed that children’s uncertainty is associated with their general psychological functioning, anxiety, depression, and psychological distress independently.

Additionally, a recent research conducted based on the findings from the time perspective research (e.g., Lewin, 1942; Nuttin and Lens, 1985), presented the possibility that IU may have a significant influence on children’s future time perspective, thus having a long-term developmental effect on the child (Ishii, 2018). The study pointed out that not being able to “determine the meaning of an illness-related event” (Mishel, 1988), or having “an incomplete or insufficient cognitive schema, or internal representation, of the illness” (Stewart, 2003), may result in children not being able to construct a more adaptive future life perspective, i.e., a more extended, valued, connected and an expectation versus fantasy future time perspective (e.g., Oettingen and Mayer, 2002; Andriessen et al., 2006). This is because future time perspective is constructed upon the individual’s appraisal of the self (for reviews on self-concept and self-knowledge; Markus, 1983; Cross and Markus, 1994) and is considered crucial for one to have a sense of control over his/her life for an adaptive future time perspective to be constructed (Husman and Shell, 2008), which IU may prevent one from constructing. The study reported that not all, but some IU, including IU experienced in the social aspect of the illness (e.g., the possibility of getting discriminated against) may affect the child’s future planning, thus suggesting management of IU may benefit children in the long run.

However, even though it has been indicated that there is a need for further investigation on the dimensions and typologies of illness uncertainties (Babrow et al., 2009), the field has not come to a conclusion regarding what uncertainties matter and what uncertainties should be dealt with for the patient’s benefit. This is especially so with children’s IU, and researchers have been claiming that some of the fundamental uncertainties children experience are still being largely overlooked (e.g., Neville et al., 2019; Pincus et al., 2018). Although the aforementioned questionnaires have facilitated research in the field, it may also have prevented researchers from examining children’s IU from the fundamental level. While questionnaires enable researchers to test hypotheses and draw conclusions on specific topics, the method is not best when researchers want to explore and describe experiences of participants (Smith, 2003). With the significant advance in medicine, the current experience of children in hospitals may well differ from the experiences 20 years ago, thus requiring a thorough, qualitative investigation of the construct once again. Additionally, previous qualitative research on children’s IU has not been conducted with long-term care in mind. As coding in qualitative research may be considered “more art than science” (Wiiliams and Moser, 2019, p.49), the researchers’ interest and goals have a crucial influence on the findings. Therefore, the goal to construct long-term care for children may lead to the emergence of new themes.

In achieving this aim, we decided to approach adolescents who have experienced pediatric cancer and have finished their treatments. Pediatric IU is an understudied topic in Japan, and to our knowledge, this is the first study to focus on pediatric IU other than Ishii (2018), who have examined survivors of different pediatric illnesses such as heart disease and cancer in Japan. In this situation, cancer survivors would be the best subject, since cancer is one of the illnesses that have been studied intensely in pediatric IU research (Szulczewski et al., 2017), therefore allowing us to deepen our analysis by comparing with previous findings.

## Materials and methods

### Methodological approach

Considering the current status of the field, we thought it best to apply directed content analysis^1^ for this research. Directed content analysis is appropriate to use when theories already exist but prior theories and research about the phenomenon is incomplete and could benefit from further description, with the goal “to validate or extend conceptually a theoretical framework or theory” (Hsieh and Shannon, 2005, p. 1281). In the present study, we aimed to explore how the framework constructed by previous studies, such as Mishel (1988), Brashers et al. (2003) could be refined or extended with new data. Thus, as explained below, we constructed our interview guide and conducted our analysis accordingly.

### Study participants

Head teacher of the in-hospital class at a university hospital (Z hospital) in Tokyo, Japan, where the first author (FA) volunteered for a year, agreed to recruit participants for the present study. Inclusion criteria included being treated at the hospital with pediatric cancer, being an adolescent (about the age 16–22), fluent in Japanese, and cognitively able to participate in the interview (as indicated by the parent). Anyone experiencing relapse were excluded from recruitment. The head teacher attempted to call the parent of every eligible former student and briefly explained about the study, and if the parent agreed to let their child know about the research, they told the child the FA’s contact information. If the child agreed to participate, he/she contacted the FA *via* email or text, and the FA explained about the study to the potential participant. Of the six females^2^ who consented to participate in the study, one canceled once due to the possibility of recurrence but found out later that it was not a relapse, and was once again included in the study on her request.

### Instruments and data collection

In 2019, the FA conducted semi-structured, in-person interviews in a private conference room at the university or off-site, as per participant preference. The participants were told in advance that the interview would last about one to 2 h, which lasted 53–171 min (*M* = 92 min). When starting the interview, the FA provided information about the study once again and obtained written consent.

Throughout the interview, the FA used an interview guide so each data would be comparable and so it would prevent the FA from forgetting any questions. We developed the interview guide in advance referring to Smith (1995) and Hsieh and Shannon (2005) (Table 1). As descripted in Hsieh and Shannon (2005), it was constructed so that broader questions were placed in the first half (e.g., about the illness and the treatment in general), and more specific questions were placed in the later half (e.g., about the experienced uncertainties). Of note, however, in order for the participants to relax and talk in a more natural way as possible, the interview guide was not strictly adhered to and was looked at only briefly for guidance. Also, especially when asking about the experience of illness uncertainty, the FA asked interpretive questions (Merriam, 1998) in order to prevent misinterpretation of the data. If participants’ explanation about their illness remained general and dictionary-like, additional questions were asked about the more specific content regarding their own treatment experience in order to capture their own experiences. Furthermore, leading questions were avoided by keeping the questions as simple as possible, and attempts were made to use the words the participants used instead of rephrasing it.

**Table 1:** Interview guide.

1	About the illness and treatment
1–1	Can you tell me about your illness and treatment?
1–2	When did you learn about the things you know about the illness and treatment?
1–3	From whom did you hear about your illness and treatment? Did you do some research yourself?
2	About illness uncertainty
2–1	Until now, were there anything you were uncertain and anxious about that you could remember?
2–2	On the contrary, were there anything you rather not know?

All interviews were audio-recorded with participant consent. Field notes and memos were made during and immediately after each interview to capture contextual observations.

### Analysis

Normally, when conducting a directed content analysis, a categorization matrix is developed based on earlier works and coding is done based on this matrix (Elo and Kyngäs, 2008). However, as Hsieh and Shannon (2005) points out, we feared that this approach may lead us to unconsciously skew the data in some way as we try to fit the data into pre-existing categories/codes. So as not to distort the data in anyway, we coded the data with the codes we defined from the data, and cross-referenced it with the existing literature after all the coding and categorization were done.

First, data from all interviews were transcribed verbatim and anonymized. All transcriptions were read repeatedly and carefully to gain a deep understanding. Then, we extracted all transcriptions that fell under the definition “the person is unable to construct the meaning of an illness related event of her/his self or another, and is aware of the state,” a definition developed to capture IU (Ishii, 2018). The extracts included phrases such as “I did not know,” “wasn’t sure,” “worried,” “why,” and “until when.” Then we coded each phrase paying great attention to the context. After coding was done, the codes were compared with previous research, especially Mishel (1988), Brashers et al. (2003), and Han et al. (2011). New and emergent themes were identified at this stage by cross referencing the codes with the data and with additional review of the literature.

Note that we analyzed the data first and translated that Japanese into English when writing this article. All translation was done by a bilingual translator and validation was done by the FA.

## Findings

In order to understand what IU children experienced and how they dealt with it, when analyzing, we took contexts very seriously, including by *whom* and in *what context* the particular statement was made in. Therefore, rather than summarizing the characteristics of the participants, we report here the background of each participant, using pseudonyms (Table 2).

**Table 2:** Characteristics of participants.

Name	Experienced cancer	Age at diagnosis and treatment	Age at interview
Mary	Germ cell tumor/Kidney cancer/Ischium tumor	13, 14	21
Sarah	Retinoblastoma/Osteosarcoma (twice)/leukemia	0, 8, 12	17
Tiffany	Acute myeloid leukemia (three times)	10, 11, 12	20
Kate	Acute myeloid leukemia (twice)	16, 18	20
Melanie	Acute lymphocytic leukemia	13	22
Jasmine	Acute lymphocytic leukemia	11	21

### Uncertainties experienced by children

Uncertainties articulated in the present research are presented in Table 3. (Below, the italicized text refers to the IU mentioned by participants.)

**Table 3:** Experienced uncertainties.

	Uncertainties told while explaining about the illness		Uncertainties told as an answer to the IU question	
	(Answers to question 1)		(Answers to question 2)	
Name	Experienced period	Experienced uncertainties	Experienced period	Experienced uncertainties
Mary	–	None	–	None
Sarah	Before treatment (second surgery)	Success/failure of the treatment	–	None
	Before diagnosed (with forth cancer)	“why me?”		
Tiffany	During treatment (third treatment)	Impact/side effect of treatment	–	None
	Now (with late side effects)	“why me?”		
Kate	–	None	At diagnosis	Prognosis (death, recurrence)
			At diagnosis	“why me?”
			Start of treatment	Safety of the treatment
			Start of treatment – now	Impact/side effect of treatment
Melanie	At diagnosis	“why me?”	Before hospital discharge	Social reaction to the illness
			After hospital discharge	Prognosis (death, recurrence)
Jasmine	When experiencing symptoms	Meaning of symptoms	After hospitalization	Structure of the hospital
	When experiencing symptoms	Correctness of the diagnosis	During treatment	Meaning/necessity of treatment
	At diagnosis	“why me?”	During treatment	Duration of hospitalization
	At diagnosis	Prognosis (death)	During treatment	Impact/side effect of treatment
	Start of treatment	Good/bad things to do during treatment	During hospitalization	How to interact with hospital staff
	Start of treatment	About the treatment	During hospitalization – several years after	Financial burden on family
	During treatment	Hospitalization itself		

#### Uncertainties related to illness and treatment

Many experienced uncertainties directly related to their illness and treatment, before, during and after the treatments.

It took time for most of the participants to receive the correct diagnosis, and Jasmine, who experienced a severe nosebleed before diagnosis, said she wondered why she was bleeding so badly (*uncertainty regarding symptom*), and after she was told that she was bleeding from using too much air-conditioning she was concerned about the correctness of the diagnosis (*uncertainty regarding diagnosis*). When finally hearing the diagnosis, Kate and Jasmine mentioned how they experienced *uncertainty regarding the possibility of death*, as Kate phrased it as “when I first heard about it, *I was worried that it was the sort of sickness people die from*.”

Most participants mentioned the uncertainties they experienced regarding their treatment. Sarah mentioned the concern she had about the second surgery (*uncertainty regarding treatment result*), which concern she did not have before the first surgery because she “did not really understand what the procedure was about at that time”:

The second time, *I was scared and anxious hearing about the procedure*…The hospital ward was not well-lighted, and I could not see anything in the dark so, well, I got anxious.

On the other hand, Tiffany, who said “*I wasn’t anxious when I was hospitalized because I knew I would be able to get back (to school),*” also talked about how she felt uncertain while receiving the third anti-cancer drug treatment:

But once, there was a time I felt sick for pretty long (during a round of anti-cancer drug treatment). *I wondered then how it was before. Like, had I always been like this*?

Kate was also uncertain about the possibility of infertility due the radiation therapy she underwent:

When I was given radiation therapy, they told me I would have less chance of getting pregnant*. I worried about becoming unable to bear a child*…

#### Uncertainties related to hospitalization

Jasmine, who was hospitalized when she was 11, talked about “uncertainty in hospitalization itself.” She first expressed her concern about how her stay would be like:

At the beginning, with so many people I didn't know, I was a bit worried, *what my stay in the hospital would be like*.

She also went on to talk about her uncertainty about the structure of the hospital:

I was anxious to go outside the hospital room… Really, I was brought to my room, and, for a long time, I was kept there, so I was really scared to go out the room.

Other uncertainties were those regarding how to interact with medical staff.

#### Uncertainty related to self

Five participants of this study, all except for Mary, talked about the “questioning” of why they had to experience cancer at least once after hearing the diagnosis, saying “why me?” “why did it come to me?”

Melanie expressed her feelings as follows:

(After hearing the diagnosis) At first, I couldn’t accept it even though I’d decided to give it my best, so I cried every day. After my treatment started, I thought “*why me*?” But anti-cancer drug treatment started when I hadn’t fully accepted the situation, so my appearances started changing, and I wore a hat all the time at first.

Sarah said “before I was diagnosed with leukemia, or maybe after, I was like “*why me?*” and Tiffany stated that “*So I do sometimes wonder why I became sick, recently”* due to her recent late side effects.

#### Uncertainty related to social response

Melanie mentioned the concern she had when being discharged from the hospital.

I wasn’t insecure in my environment at the hospital, but after going back to school, I could tell everyone was being extra nice. *I hadn't told them about my wig, so that made me insecure and anxious (about how they would react)* …So there was some talk, like how my hair doesn't flutter in the wind [laughs]. Of course, no one said that to my face, so the wig stayed a rumor.

She found out about this rumor by asking a close friend. The reason she had kept her illness and her wig a secret to everyone was because her parents suggested not to tell anyone. After moving into senior high school, like her parents, she also strongly felt like “I do not want anyone to know.” Entering senior high school, where no one knew her past, enabled her to feel like she had become a “new me.”

### Managing illness uncertainties

Even when asked directly, some of the participants did not mention what they did to manage the uncertainty they experienced, suggesting that time was what helped the uncertainty to resolve. This was so with Sarah’s concern about the surgery result, the uncertainty Tiffany experienced about the effect of the treatment, the uncertainty Melanie experienced in regard to the prognosis.

#### Reducing uncertainties by talking with medical staff

Some of the participants mentioned that they asked questions when they had any concerns. Mary, who mentioned she “*did not really have any*” uncertainties, said “*I asked them to tell me everything*” and said:

I was allowed to ask questions. They would answer me…Residents are just below 30, close enough to be my older sisters, so they would even help me with my studies and tell me about my illness. I’d ask them things like “How long would it be till I can go home?”

Kate also mentioned that she dealt with her uncertainties by “*asking the doctor, writing down everything he answered.*”

Jasmine, who did not know how to interact with physicians, also mentioned that some of the uncertainties she experienced were reduced as a result of the interaction with physicians. She said:

It felt so weird (to have the catheter in) and *I wasn't sure if it was okay for me to move around*… So I was in bed for a week like this [places both arms tightly at her sides]. The doctor touched my shoulder and realized it was really stiff…I asked if it was alright if I moved, and he replied “of course.”

#### Reducing uncertainties by gaining written information

Melanie mentioned about the pamphlet a nurse gave her that said “About leukemia,” which “describes the disease briefly, and how it’s curable, so we should all fight together to overcome,” suggesting that it gave her information to reduce the uncertainties she was experiencing after hearing the diagnosis.

#### Reducing uncertainties by seeing others

Jasmine experienced uncertainty with regard to the possibility of death when she was diagnosed, and in addition to the explanation from the physicians, the appearance of children who were hospitalized in the ward with the same illness helped resolve this uncertainty.

*Witnessing various cases (of children with the same disease)*, and hearing the doctor say that with medical care advancing each year, it is no longer an incurable disease but one that is easy to cure, my worries gradually melted away and I felt relieved, yeah, something like that. *(Being in a private room was hard) because you have little information. You don't get to interact with others*.

Melanie also said that when she was thinking “why me?” she saw other children’s “*positive attitudes*” and thought “I just gotta do this.”

#### Accepting uncertainties

Kate, who experienced acute myeloid leukemias twice, said she was experiencing uncertainties about the prognosis and why she had to experience the illness:

When I talked to my doctor, he looked me straight in the eye and said “I'll do my best so that you can become a wonderful adult, so let’s do our best together.” That’s when I thought, wow, he’s a great doctor, *maybe I might be alright.* (Kate)

The physician’s words did not necessarily have the scientific information that might reduce Kate’s IU, however, Kate still says that the conversation with her doctor convinced her that things might turn out okay. In her case, the uncertainties Kate had been experiencing before interacting with the particular doctor became less of a concern after that interaction. This phenomenon will be further discussed later.

## Discussion

The aim of this study was to examine the illness uncertainties (IU) that children experience and the ways they manage them by interviewing six female adolescents who treated their cancer at least once at Z hospital. As has been theorized and reported in previous research, the participants have experienced various uncertainties regarding their illness and treatment, such as uncertainties regarding the prognosis of the disease, as well as the side effects of the treatments (Mishel, 1988), and the social reaction to the illness (Brashers et al., 2003). On the other hand, some of the uncertainties mentioned in the present interview have not been discussed in previous research. We should also point out that, overall, the amount of uncertainties mentioned in our interview was not a lot, with half the participants saying “none” to the question asking them what uncertainties they experienced.

Since the main aim of our study was to extend previous research and to gain knowledge that would be of use when constructing care for children, in the following section, we focused on discussing themes and ideas that literature review indicated as supportive in achieving the above goal, even if they were not commonly experienced by all participants.

### IU experienced by children

#### Uncertainties related to hospitalization

We believe that one of the typologies of uncertainty that emerged in the present study is the uncertainties related to hospitalization. Surprisingly, this type of uncertainty has not been covered in the context of IU research (refer to following for list of uncertainties covered in previous research; Stewart, 2003; Pai et al., 2007; Stewart et al., 2010), maybe because research has not progressed to the point where uncertainties experienced by relatively young children are fully revealed. Nevertheless, in related research, it has long been reported that younger children also experience anxiety and stress due to hospitalization and treatment (e.g., Pearson, 1941). For instance, of the 90 children from ages four to 6 years who were interviewed for hospital-related fears, 26% reported they were scared of being hospitalized and being in the hospital (Salmela et al., 2009). The study also reports that a certain number of children were afraid of the hospital’s physical environment (buildings and machinery; over 21%) and unfamiliar people (10%). These research findings in the related fields indicate how natural and reasonable it is for Jasmine to have experienced the uncertainties she reported.

Researchers have claimed that illness experience is one of the most important experiences in the socialization of children in terms of learning about physical disorders, how to deal with symptoms and medical services (e.g., Parmelee, 1986). However, from the above findings, if the child has not acquired or is in the process of acquiring knowledge about hospitals and treatments, it is natural that the child would be experiencing uncertainties regarding it in addition to other common uncertainties regarding illness and treatment.

#### IU with answer/IU without answer

This research also indicated the need for researchers and medical workers to be sensitive to the characteristics of the uncertainties experienced by children; whether or not they have answers in science.

##### Uncertainties regarding his or her case

Sarah, who said she was “explained in detail” and thus “knows” about the illness, also said she had experienced uncertainty about how the surgery would result. Kate also said she was well-explained. However, to this day, she is still experiencing an uncertainty regarding the possibility of infertility due to the treatment. What these uncertainties have in common is that they are uncertainties of “what would happen *in my case*” that remain even after information about the general possibilities are provided. These narratives indicate that there are critical differences between the objective, scientific, general information and the information regarding “in his or her case.” This IU includes, but is not limited to, the IU Stewart (2003) reports as *not being able to predict*.

In other fields, two types of uncertainty have been clearly distinguished. In medical sociology research, the uncertainty caused by one’s incomplete or imperfect mastery of available knowledge, and the uncertainties that stems from the limitations in current medical knowledge is recognized (Fox, 1980). Furthermore, in communication science, Babrow (2001) distinguishes two uncertainties in his problematic integration (PI) theory; epistemological uncertainty and ontological uncertainty. Epistemological uncertainty refers to ways of knowing; it is uncertainty about too much, too little, or inconsistent information, whereas ontological uncertainty refers to uncertainty about “the nature of the world.” Matthias (2009) empirically examined the uncertainties experienced by pregnant women based on PI theory and reported that the effective support may differ depending on whether the experienced uncertainties are ontological or epistemological, indicating the possibility that the way to resolve these two types of uncertainties may vary.

However, as can be seen in the definition of IU as “caused by lack of information” (Mishel, 1984), Mishel’s and the following studies, which started from studying uncertainties experienced by patients with acute illness, have focused on dealing with IU that can be reduced with sufficient information, in other words, “uncertainties that have answers somewhere.” After IU experienced by patients with chronic diseases have also started to get investigated (e.g., Brashers et al., 2003), IU that does not necessarily have an apparent answer, such as the social impact of the illness, have become subject of research. As stated above, Stewart (2003) has also reported that some of the children with cancer acknowledged the inherently unpredictable nature of the illness, pointing out that the unpredictability of the illness could not always be resolved with information and experience.

##### Why me?

We should also discuss here about the uncertainties that five of the participants mentioned; the “questioning” of why they had to experience cancer. Everyone except for Mary mentioned this, saying they thought “why me?” “why did it come to me?” at least once after hearing the diagnosis.

The word “why,” while taking a form of question, may merely express emotions such as strong anger, sadness, and shock rather than one really wanting to know the cause (e.g., Abela et al., 2002; Katsumata, 2015). However, at least for some individuals who have experienced stressful life events such as illness or injury, it is indicated that the expression “why me?” represents a kind of incomprehension, and that there are processes individuals go through answering to this question. For example, examining the experiences of patients with fibromyalgia, Madden and Sim (2006) described the process by which patients experience illness as the process of confronting the uncertainty of the cause of the illness, that is, the process of answering the question “why me?” The study suggests that while the biomedical explanation provided at the hospital could be of help to resolve this uncertainty, it is not enough, and it is an uncertainty for the individual to resolve by finding meanings.

Including this questioning of “why me” and looking at the other uncertainties discussed in this and previous studies, it is strongly suggested that patients and their families are experiencing many unanswerable IU, such as uncertainties one might experience even with sufficient objective and scientific information, “Can *my* illness be cured (not in general terms)?” “What will happen to *my* future?” (e.g., Hilton, 1994; Madden and Sim, 2006). Whether or not the uncertainty has an answer in science and objective information may be a significant factor when supporting children with managing IU.

### Managing illness uncertainties

#### “Role model” as information to resolve IU

Many studies have attempted to find strategies to reduce patients’ and families’ uncertainties, and many studies have also examined and revealed the effectiveness of peers, but no studies have considered the mere existence of peers as means to mitigate the experienced uncertainties. Nevertheless, the present study suggests this may be a useful strategy.

Many approaches have been reported as effective in reducing uncertainties experienced by patients and families. For example, in adult studies, written materials have been used to reduce IU and their effectiveness have been reported (e.g., Zhang et al., 2020). In the context of other research, the effectiveness of preparation (explanation of the illness and treatment) using visual aids has been suggested as a means of relieving anxiety in children (Brewer et al., 2006). In fact, in this study, for example, Melanie mentioned the pamphlet “About Leukemia” distributed by nurses, suggesting that the information obtained there was useful.

The effectiveness of peers has been pointed out in many studies as well. Peer support has been shown to have effect in gaining live information and emotional support (e.g., Dunn et al., 2003). In the present study, Mary said that she was able to talk about hair loss because girls of close age were in the same room, and Melanie also mentioned that she talked about wearing wigs with other girls she shared room with, suggesting the two had benefited from the support they had from peers experiencing similar illnesses and treatment processes.

In contrast to the above research on peers, in Jasmine’s and Melanie’s case, merely the existence of peers, those who were able to overcome a similar adversity, seemed to have been informative. Jasmine, who said she could not read the distributed pamphlet because she was “scared of reading it,” said that seeing other children in the ward helped her reduce the uncertainty she was experiencing. This is close to what Rebeiro et al. (2016) depicted as *society’s mirror*, which is listed as one of the essences identified by peer support workers about the service they provide. In their article, they describe how peer support workers provide “a mirror reflection” to mental health users that recovery is possible, bringing them hope. These episodes and reports demonstrate the informative value peers have that may help one reduce their IU. Especially for young children whose use of written materials is limited and children who gradually become independent from their parents, the information obtained from “the appearance of peers,” or a role model, may be particularly beneficial.

### Relationship with medical staff and uncertainty acceptance

For nearly 20 years, research regarding IU has been conducted on the implicit premise that IU should be reduced in order to increase the individual’s well-being. This changed after Brashers (2001) made a proposal stating that uncertainty does not necessarily induce anxiety, which led researchers to develop theories in recognition of the fact that people sometimes do not seek to reduce uncertainty. This includes the UMT (uncertainty management theory), advocated by Brashers and Hogan (2013), which theorized how people use information to reduce, maintain, and increase uncertainties.

The data from the present study suggest that reducing, maintaining, and increasing their IU may not necessarily be the only goal people strive to reach in the face of uncertainty and *uncertainty acceptance* may be one form of solution. In a recent study that examined specialists’ influence on parental uncertainty (Kerr et al., 2019), it is reported that the established confidence and trust between parents and specialists have a great impact on the uncertainty parents experience. Based on an interview conducted on 29 parents who have children with complex chronic illness, Kerr et al. (2019) states that “when parents expressed trust, regardless of whether it was inherent or developed during the visit, their perceptions of the team of specialists as credible directly affected uncertainty, making it less threatening.”

Kate’s case and the report from Kerr et al. (2019) indicates the importance of physicians and specialists, suggesting that the relationship with these medical staff members may influence the individual’s perception of IU. In both cases, the uncertainties are not reduced with information, nor are they maintained or increased. Rather, as Kerr et al. (2019) put it as “less threatening,” the IU simply became less of a threat and concern to the patient and their parents. Considering that the uncertainty has not changed but does not bother the individual anymore, this phenomenon may be described as *uncertainty acceptance*, rather than the IU being reduced or managed (Brashers and Hogan, 2013). We still do not know which IU can be accepted, and which medical staff members could play the crucial role. Considering how we have presented IU without answers and how it may be difficult to reduce them by objective information, the idea of acceptance may be of great significance to be examined in the future.

## Limitations of the present study

Several limitations of this study are noteworthy. First, we should note here that our data was collected from participants who were able to overcome the difficult, sometimes traumatizing experience enough so that they were able to tell their story to the FA. One potential participant did not contact the FA even after she was given the contact information from her mother. We do not know the reason she decided not to participate; she may not have had the time or maybe she wasn’t ready to talk about her experience yet. Thus, we do not believe we have covered all aspects of IU, and our findings may not necessarily be generalizable to female cancer survivors more broadly.

Second, as mentioned above, most of the participants in the present study had leukemia, which is the most famous pediatric cancer in Japan. Unlike other diseases (Madden and Sim, 2006), leukemia has less ambiguity in terms of diagnosis but is a disease with “moral” and “incurable” image (Furuya, 1998). Therefore, there may be completely different findings if different, not well-known diseases were targeted. Third, the participants were recruited from Z hospital, which is one of the most prestigious hospitals in Japan. Therefore, even if it wasn’t mentioned in the interview, it is very likely that participants had great trust on the hospital and physicians, which may have a significant influence on the uncertainties they experience (Kerr et al., 2019).

## Data availability statement

The datasets presented in this article are not readily available because the confidentiality of participants may be compromised and consent was not obtained from participants for raw data to be made available. Each person’s interview in brief, including the uncertainties and management episodes, together with the statements that characterizes the person in detail is presented in the Supplementary material. Requests to access the datasets should be directed to YI, yuishii@p.u-tokyo.ac.jp.

## Ethics statement

The studies involving human participants were reviewed and approved by the University of Tokyo Research Ethics Committee of University of Tokyo and approval by the Dean of the Graduate School of Education of University of Tokyo. Written informed consent to participate in this study was provided by the participants and the participants’ legal guardian/next of kin for participants under the age of 18.

## Author contributions

YI conducted the research and wrote the manuscript. TE provided critical revisions of the draft. All authors contributed to the article and approved the submitted version.

## Funding

The authors disclosed receipt of the following financial support for the research, authorship, and/or publication of this article: This work was supported by the JSPS KAKENHI [15J12105].

## Conflict of interest

The authors declare that the research was conducted in the absence of any commercial or financial relationships that could be construed as a potential conflict of interest.

## Publisher’s note

All claims expressed in this article are solely those of the authors and do not necessarily represent those of their affiliated organizations, or those of the publisher, the editors and the reviewers. Any product that may be evaluated in this article, or claim that may be made by its manufacturer, is not guaranteed or endorsed by the publisher.

## Footnotes

^1^An anonymous reviewer suggested that the analysis done in this study might be thematic analysis and that the analysis process is very similar to what is described by Braun and Clarke (2006). Thematic analysis is a method for identifying, analyzing and reporting patterns (themes) within data (Braun and Clarke, 2006). In fact, upon examination, they are indeed very much alike. Therefore, for our readers’ better readability, for a moment, we thought of rewriting the analysis section. However, as Braun and Clarke (2006) persists, the researchers’ assumptions, theoretical positions and values in relation to qualitative research has a significant impact on their reports. Thus, we decided to keep the original description, since that is in fact what we have done, and we felt that it would be wrong to alter it by concealing the fact that we originally applied directed content analysis with the goal to "to validate or extend conceptually a theoretical framework or theory" (Hsieh and Shannon, 2005, p. 1281). It should be noted, however, that the analysis we conducted is closer to, and almost identical to the idea and method of thematic analysis described by Braun and Clarke (2006).

^2^The present study did not intend to recruit only female participants but it happened as a result.
