# Supplementary material for: Reconsidering children’s illness uncertainty and management experiences with female Japanese cancer survivors
Source: Front Psychol. 2022 Nov 23;13:1006267. doi: 10.3389/fpsyg.2022.1006267 (PMC9727163; doi:10.3389/fpsyg.2022.1006267)
Supplement: Supplementary file 1 [file Table_1.DOCX]

Supplementary Material

# Supplementary Data

**Mary’s case.** Mary realized something was wrong with her the month after she entered junior high. She had a high fever, and after taking medicine that was prescribed by her primary care doctor who thought the fever was to due constipation, the fever did not go down, so went to a university hospital, where she found out her ovary was at high risk of rupture and had to undergo surgery that day. She says she doesn’t remember anything about that day because everything went so fast. After the surgery, she was told that they found a tumor in her kidney as well. She had experienced pain in her hip before, but had thought the pain was caused from her bad posture, never suspecting it was caused by cancer.

When asked about how she came to know about the details about her illness and treatment, she answered as follows:

*I asked them to tell me everything*. Also, I sat through all the explanations on the surgery and treatment. You know how data on blood and stuff are all written in alphabet? So there’s all these data on a piece of paper, but the doctors at the Z university explained to me like, this indicates white blood cell levels and its usually around this high but in my case it’s this much, and all that. They were so nice. *And their explanation was easy to understand* [laughs].

When asked if she have had any concerns or uncertainties, she said she have had “*none*.”

*I didn't really have any* [laughs]. I was allowed to ask questions. They would answer me...Residents are just below 30, close enough to be my older sister, so they would even help me with my studies and tell me about my illness. I’d ask them things like “How long would it be till I can go home?”

This statement indicates that she had experienced IU but was able to resolve them naturally by asking residents who were close age.

**Sarah’s case.** Sarah said they found cancer in both her eyes and had no choice but to remove the right eye, but “was just barely able to leave and treat the left eye with radiation.” When she was in second grade, she got second cancer in her bone near her left eye due to the radiation treatment. It was her father who noticed that her left eye was popping out. She said, “we wanted to leave the left eye, so we scrapped off the cancer part of the bone and put the remaining part back inside, but it recurred so we just had to take the whole bone out.” As for the fourth cancer, leukemia, she talked about how her nosebleed did not stop on the night of the day she took an entrance exam for junior high school. She had lost her appetite and her joints had hurt for a while, but she thought all of it were caused by the stress of the exam.

In the interview, Sarah spent a lot of time talking about the osteosarcoma.

When I was losing my sight, I guess that was one of the hardest times. Losing sight when I was once able to see was, um, pretty, I don't know, really hard I guess.

She also mentions the concern she had about the second surgery (*uncertainty regarding treatment result*).

When hearing about my surgery, the first time I would still have an eye left, so I wasn't really that scared. I went to the surgery room laughing saying “See you later!” [laughs]. But, I guess, I didn't really understand what the procedure was about at that time…The second time, *I was scared and anxious hearing about the procedure*…The hospital ward was not well-lighted, and I couldn't see anything in the dark so, well, I got anxious.

Also, she talked about the “*question*” she had, saying “before I was diagnosed with leukemia, or maybe after, I was like “*why me?*” (*uncertainty regarding self*). She said she never talked about this with anyone, and especially not with her doctor-in-charge, because she felt like he would just lightly say “you’re fine, you’re fine!” She went on to say “*I used to think I got it because my grandpa did too, but it doesn't matter now [laughs],*” saying “*If I get sick again, all I need to do is treat it again, so I’m good as long as I’m living*.”

In terms of how she learned about her illness, she said her mom wasn’t “the type to hide,” and that while she was hospitalized with osteosarcoma or leukemia, her mom told her about the disease and explained details. Thus, regarding the uncertainties she experienced, her answered “*I don't think I have had anything I’m uncertain of*.”

**Tiffany’s case.** Tiffany found out about her leukemia when she was in fourth grade. It was found by a blood test she took based on her parents’ suggestion, who thought her lips were too pale. Soon after, she was hospitalized at a children’s hospital.

In her interview, she talked mostly about her concern about her studies. Stating that since there wasn’t an in-hospital school at the children’s hospital, she said “I pretty much did my studying by myself.” After seven months of anti-cancer drug treatment, she was discharged the following summer, but was hospitalized again at Z hospital due to recurrence. During this period, she said she not only did anti-cancer drug treatment and radiation therapy, but also had her sister’s bone marrow transplanted which was “a full match.” However, her cancer once again relapsed, “because the GVHD was not effective enough” in her case. During the next hospitalization, which started when she was in six grade, she had a bone marrow transplant again, on the first day of junior high school.

Tiffany says she heard about the diagnosis the day she was hospitalized. She also heard that she would be hospitalized for a long period and that she would be getting treatments, but says she did not learn about the specifics of the treatments.

I’d only heard that it would make me feel sick. And that my hair would fall out. I didn't ask about how long it would take, or details. I think they did say about how many months. But I don't remember asking about it myself, really.

When the FA asked her about the uncertainties she experienced, she said “*I wasn’t anxious when I was hospitalized because I knew I would be able to get back (to school).*” However, she also mentioned the following, indicating she had experienced *uncertainties regarding treatment*.

But once, there was a time I felt sick for pretty long (during a round of anti-cancer drug treatment). *I wondered then how it was before. Like, had I always been like this*?

Also, she talked about how she has been dealing with the late side effects of the treatment she received, and how she has recently been questioning why she became sick (*uncertainty regarding self*).

I'm having problems with my eyes and am taking medication (recently). It has nothing to do with my sickness, but I've been to the ophthalmologist a lot. *So I do sometimes wonder why I became sick, recently*.

**Kate’s case.** Right at the very start of the interview, Kate started explaining about her cancer in detail, saying “I had Acute myeloid leukemia, and the subtype of M0.”

When she was 15, she was experiencing strong fatigue and her legs had swollen up. She saw a few physicians and later found out that she had leukemia at Z hospital. Kate described about her treatments in detail as well.

My treatment, well, it was my first so we took it slow. I think we started off with a week or something. One week with an anti-cancer drug which everyone takes called Cytarabine, and, another colored anti-cancer drug. It was blue and looked unpleasant. That made me feel really sick for the first time.

So, I had five rounds of anti-cancer treatment. I did one week, and then I got a month off. The second round was orange instead of blue, and I didn't get sick. I was fine the second, third, fourth and fifth round, but around the third round, oh, no, it was the first. The first time, I had a bone marrow aspiration, and they told me that the drug wasn't working on the bad cells so I needed a transplant, but we went ahead with the five rounds. Plus, the transplant, and then radiation treatment. Also there was one type of anti-cancer drug different from the usual kind, which flowed with double the speed. Um....I think I had radiation therapy three times, maybe? Three times. Twice a day, three times, I think. And I had the transplant on October 30th, Halloween.

After all the treatment was done and she was discharged, the cancer relapsed. She found this out at a follow-up check and she chose to change the hospital to where her doctor-in-charge had relocated to. Whenever she had treatment, one week before it, she was called in with her mother to hear the details and “signed consent forms if there were any.”

When asked about the uncertainties she experienced, she first mentioned the possibility of death (*uncertainty of the prognosis*), saying “when I first heard about it, *I was worried that it was the sort of sickness people die from*.” She then continued to talk about an uncertainty regarding the possibility of infertility due to the treatment, which she is still experiencing today (*uncertainty of side effect of treatment*).

Another thing I was worried about was, when I was given radiation therapy, they told me I would have less chance of getting pregnant*. I worried about becoming unable to bear a child*…

Regarding this uncertainty, she asked the doctor, who told her that “even though they say that radiation therapy might affect fertility, I’ve seen more people who got pregnant.” However, she continued by saying,

*But I’m taking medicine to induce menstruation, I need medicine to induce menstruation, I’m worried if I’m really gonna be able to get pregnant*.

She also mentioned how she wondered why the cancer came to her (*uncertainty regarding self*).

A year before I was hospitalized, when I was in ninth grade, my classmate’s brother had gotten leukemia and died. So, I was scared....*I wondered why it came to me*.

When she was experiencing uncertainties about the prognosis and why she had to experience the illness, she had an interaction with her doctor-in-charge, which made the uncertainties less of a concern for Kate.

When I talked to my doctor, he looked me straight in the eye and said “I'll do my best so that you can become a wonderful adult, so let’s do our best together.” That’s when I thought, wow, he’s a great doctor, *maybe I might be alright.* (Kate)

Finally, she talked about her concern about the safety of the blood transfusion (*uncertainty of the safety of a treatment*).

When I had the blood transfusion, right at the time I was in the hospital, there was talk about how the blood for the transfusion…the person who had donated the blood had HIV and the person who received it had gotten it. *I thought “What? It isn’t safe?*”.

She dealt with this by “*asking the doctor, writing down everything he answered.*”

**Melanie’s case.** Melanie had played basketball since she was in elementary school, but after she started junior high, she realized her physical strength had dwindled significantly. Her primary doctor suspected Osgood-Schlatter disease but her basketball coach, who recently had his wife diagnosed with cancer, recommended her to go to a bigger hospital.

By the time she was at the hospital she was barely able to walk, and after some tests, she was transferred to Z hospital and “had to stay there.” The test results came out soon.

A whole bunch of doctors came in (to my hospital room), and while I was wondering what was happening, my dad started crying…So I was convinced something was wrong by the time the doctor, he was the straightforward type, told me. There was no beating around the bush, and he said “You have been diagnosed with ~.” *I wasn't familiar with the details of the disease, but because I had heard of the name on television, tears welled up the second I heard the diagnosis and I thought I was going to die*.

It took time for her to accept her situation and she always wore a hat and never wanted to take a bath. However, seeing other children in the same ward slowly changed her mind.

By the time my head started to itch, I was gradually able to accept, and in the end, I was like “Fine, I'm going to go take out all my hair today.” My parents persuaded me too. *And the other kids, the ones who’s been in the hospital for a while, were doing okay, so seeing them be optimistic made me feeling like I just gotta do it*.

She also talked about the pamphlet a nurse gave her, which “describes the disease briefly, and how its curable, so we should all fight together to overcome,” and also mentioned how she was explained about the treatments from doctors, while also mentioning that she “*asked everything she was concerned about*.”

When asked about the uncertainties she experienced, she first talked about her experience after discharge (*uncertainty of prognosis*).

We were told that we couldn't be sure that I was fully cured until five years had passed since being discharged from the hospital and treatment. So, my parents were scared for those five years, but I didn't take it seriously at all…Well, I rebelled against my parents and said stuff like “Leave me alone” and “I'm fine!” My parents, crying, I don't think they had planned to, but warned me how this girl or that boy was no longer with us; that I have to be careful or I’d get pneumonia and end up back in the hospital. *So I had an image of being hospitalized again as meaning the end, and was scared for a while after being discharged*.

She said this uncertainty faded away after five years from discharge.

She also talked about her wig (*uncertainty regarding social response*).

I wasn't insecure in my environment at the hospital, but after going back to school, I could tell everyone was being extra nice. *I hadn't told them about my wig, so that made me insecure and anxious*…So there was some talk, like how my hair doesn't flutter in the wind [laughs]. Of course, no one said that to my face, so the wig stayed a rumor.

She found out about this rumor by asking a close friend. The reason she had kept secret about the wig and about her illness to everyone was because her parents suggested not to tell anyone. After moving into senior high school, like her parents, she also strongly felt like “I don’t want anyone to know.” Entering senior high school, where no one knew her past, enabled her to feel like she had become a “new me.”

**Jasmine’s case.** Jasmine found out about her illness when she was in fifth grade. She felt dizziness when her relatives were gathered for a summer festival, and soon after, she had a severe nosebleed. She wondered why she was bleeding so badly (*uncertainty regarding symptom*), and after she was told that she was bleeding from using too much air-conditioning she was concerned about the correctness of the diagnosis (*uncertainty regarding diagnosis*).

At an otorhinolaryngology clinic she went after, she was suggested to take a blood test. After the result came out, the doctor called her mother to tell her to go to a bigger hospital, and at the Z hospital, she was told to hospitalize.

Jasmine then talked about the time she was diagnosed, indicating that that was the hardest period for her throughout the experience. She here talks about how she wondered the cancer happened to her (*uncertainty regarding self*).

About a week after I was hospitalized my family was summoned and explained to first. That their child had leukemia, and what type it was. After that it was my turn to be told, and *I wondered why this happened to me*, I couldn't accept it. *Yet, I felt my family must be having a harder time so I kept from crying, but the doctor must’ve noticed; he said it’s okay to and tears streamed down.* Yeah, that part was a bit hard. Then talk turned to the treatment. Before starting the treatment, they explained I would need to plied with a lot of medicine to fight it, and there will be side effects, but that I could do it.

Around the same time Jasmine was hospitalized, a famous actress in Japan died from leukemia, making her concerned about the possibility of death (*uncertainty regarding prognosis*).

The doctor told me I would be cured but the possibility of death wasn't zero. Having heard that there were some cases in which patients died gave me something to think about.

Regarding this uncertainty, she indicated that in addition to the explanation from the physicians, the appearance of other children who were hospitalized in the ward with the same illness helped resolve it.

*Witnessing various cases (of children with the same disease)*, and hearing the doctor say that with medical care advancing each year, it is no longer an incurable disease but one that is easy to cure, my worries gradually melted away and I felt relieved, yeah, something like that. *(Being in a private room was hard) because you have little information. You don't get to interact with others*… Getting sick in 5th grade puts you in a position with little information. You have limited means of gathering information.

Regarding treatment, she mentioned the concern she had when she was placed an arterial catheter (*uncertainty regarding treatment*).

It felt so weird (to have the catheter in) and *I wasn't sure if it was okay for me to move around*. Even just moving my shoulders like this, I thought the tube would snap. So I was in bed for a week like this [places both arms tightly at her sides]. The doctor touched my shoulder and realized it was really stiff…I asked if it was alright if I moved, and he replied “Of course.”

She also said her parents received “a piece of paper” that had explanation about the treatments written on it, and that she could have taken a look at it but “didn’t look.” Rather, she’d “wonder what medicine they are injecting” and “what kind of side effects they could cause.” (*uncertainty regarding treatments*).

She also talked a lot about how it took time to get familiar to “living with an IV,” how she wasn’t able to communicate well with nurses. Including the following statement, it is indicated that she was experiencing *uncertainty regarding hospitalization itself*.

At the beginning, with so many people I didn't know, I was a bit worried, *what my stay in the hospital would be like*.

When asked about the uncertainties she experienced, she first mentioned the *uncertainty she had about the structure of the hospital*, and once again stated that she didn’t know how to interact with hospital staff (*uncertainty regarding life in hospital*). She also said she didn’t know why she had to do some of the treatments (*uncertainty regarding meaning of a treatment*). She also mentioned the following.

Simply, *I was anxious about when I could get out*, really. The period wasn't exactly fixed, it would change, so I was worried about the progress. Depending on how I was healing, if it wasn't going well the doctor would try a different type of medication, and if that didn't work out either then he would reconsider…And there were actually times that my stay got extended. My pancreas got bad because of a side effect and I was temporarily diabetic…and it pushed back my discharge about a month I think.

This statement implies that she wasn’t concerned about the general length of hospitalization that is required, but rather, how things would turn out in her case. In fact, this statement also indicates that she knew well about how her hospitalization period could be affected by her status and how her hospitalization actually got extended due to the status of her pancreas.

Finally, she talked about the concern she had about “money.”

I was concerned about the cost of my hospitalization, like how much it cost for a day in bed, or how much a single meal cost. And sometimes there was this special lunch menu where if you apply for…It was a bit of an upgrade from the normal menu, and was offered maybe once a week. And occasionally when I applied for that, I’d get this piece of paper stating the amount I have to pay, the difference from the normal menu, which made me go ahhhhh. It made me wonder, if getting an upgraded menu cost this much, then how much does a normal hospital menu cost?

She wasn’t able to ask her family about this for a while. When she and her family participated in another study several years after discharge, they decided to “sort out the fact.” This was when she “worked up the courage” and asked, to which they answered by saying insurance and financial assistance from the prefecture covered a lot.
